# WIF1 causes dysfunction of heart in transgenic mice

**DOI:** 10.1007/s11248-013-9738-z

**Published:** 2013-08-07

**Authors:** Dan Lu, Wei Dong, Xu Zhang, Xiongzhi Quan, Dan Bao, Yingdong Lu, Lianfeng Zhang

**Affiliations:** 1 Key Laboratory of Human Disease Comparative Medicine, Ministry of Health, Institute of Laboratory Animal Science, Chinese Academy of Medical Sciences and Comparative Medical Center, Peking Union Medical College, Beijing, People’s Republic of China; 2 Key Laboratory of Human Disease Animal Model, State Administration of Traditional Chinese Medicine, Institute of Laboratory Animal Science, Chinese Academy of Medical Sciences and Comparative Medical Center, Peking Union Medical College, Building 5, Panjiayuan Nanli, Chaoyang District, Beijing, 100021 People’s Republic of China

**Keywords:** WIF1, Heart, Transgene, Mice

## Abstract

Wnt activity is a key regulator of cardiac progenitor cell self-renewal, differentiation and morphogenesis. However, Wnt inhibitory factor 1 (WIF1), a antagonists of Wnt signaling activity, its potential effects on heart development has not yet been approached by either in vivo or in vitro studies. Here, the expression of WIF1 was regulated in a different way in the dilated and hypertrophic cardiomyopathy heart from transgenic mice by mutations in cardiac troponin T, cTnT^R141W^ and cTnT^R92Q^. The heart tissue specific transgenic mice of WIF1 was studied using M-mode echocardiography and histologic analyses. Production levels of an array of effectors and transcription factors that impact cellular organization and tissue morphology were measured. The effects of WIF1 on β-catenin pathway could be reversed by LiCl regarding signaling pathways and effector and respondent molecules in H9c2 cells, consistent with the expression levels of c-myc, natriuretic peptide precursor type B and skeletal muscle actin α1. Among the most noteworthy findings were that WIF1 impaired the function and structure of heart, and the effects on β-catenin pathway maybe the course of the former. It is anticipated that our findings will contribute to expansion of our understanding of WIF1 biological function on heart development and possible modes of treatment of heart diseases.

**Electronic supplementary material:**

The online version of this article (doi:10.1007/s11248-013-9738-z) contains supplementary material, which is available to authorized users.

## Introduction

Wnt is a key regulator of cardiac progenitor cell self-renewal, differentiation and morphogenesis (Cohen et al. 2008). Various secreted Wnt antagonists interact directly or indirectly to affect Wnt signaling and influence its regulatory processes (Kawano and Kypta 2003), and their modulating capacities are thus important in cardiac development. Wnt signaling can be mediated through either canonical β-catenin–mediated Lef/Tcf transcriptional activity or other noncanonical pathways (Macdonald et al. 2007; Semenov et al. 2007). Extracellular antagonists of the Wnt signaling pathway include the secreted frizzled-related protein family, Wnt inhibitory factor 1 (WIF1), and the Cerberus and Dickkopf (Dkk) family.

Wnt inhibitory factor 1 was first identified as an expressed sequence tag from human retina with highly conserved among sepecies (Hsieh et al. 1999). The WIF1 binds directly to extracellular Wnt ligands, preventing their interaction with the receptors and leading to β-catenin degradation (Hsieh et al. 1999). WIF1 is present across vertebrate families and consistes of an N-terminal secretion signal sequence, the WIF domain, five EGF-like domains and a hydrophilic C terminus (Malinauskas et al. 2011). Human WIF1 binds through to eight Wnts [3a,4,5a,7a,9a,11 (Surmann-Schmitt et al. 2009), wingless and *Xenopus* Wnt8] and a protein involved in neuronal differentiation, olfactomedin 1(Nakaya et al. 2008).

During embryogenesis in *Xenopus* and zebrafish, expression of WIF1 is first detectable at the start of somitogenesis in the paraxial mesoderm, and WIF1 expression continues in adults in the heart, lungs and cartilage-mesenchyme interfaces of various species (Surmann-Schmitt et al. 2009). Moreover, WIF1 play an essential role in the regulation of Wnt signals in development of central nervous system (Hu and Zhao 2010) [9], and it is indicated that WIF1 enhances cardiomyogenesis (Buermans et al. 2010).

The downregulation of WIF1 by promoter hypermethylation has been reported in various human malignancies including carcinoma of urinary bladder, lung, brest, esophagus and stomach (Ding et al. 2009; Urakami et al. 2006; Mazieres et al. 2004; Ai et al. 2006; Clément et al. 2008; Chan et al. 2007; Taniguchi et al. 2005). In addition, it has been shown that WIF1 functions as a tumor suppressor in melanoma, nasopharyngeal, esophageal, stomach, brest, and lung cancers (Clément et al. 2008; Chan et al. 2007; Taniguchi et al. 2005; Lin et al. 2007, 2008; Kim et al. 2007; Wissmann et al. 2003), and overexpression of WIF1 inhibites the growth of cells from lung and bladder cancers (Tang et al. 2009).

We reported previously that cTnT^R141W^ transgenic mice manifest progressive chamber dilation and contractile dysfunction, and have a pathologic phenotype similar to that of human dilated cardiomyopathy (DCM) (Watkins et al. 1995; Juan et al. 2008). In addition, cTnT^R92Q^ transgenic mice manifest ventricular wall hypertrophy, reduced ventricular chamber and diastolic dysfunction, and have a pathologic phenotype similar to that of human hypertrophic cardiomyopathy (HCM) (Thierfelder et al. 1994; Tardiff et al. 1999; Javadpour et al. 2003). Interestingly, the expression of mutant cTnT^R92Q^ and cTnT^R141W^ genes causes divergent pathologic phenotypes, as reported for both the human disorder and mouse models of cardiomyopathy (Watkins et al. 1995; Thierfelder et al. 1994; Tardiff et al. 1999; Javadpour et al. 2003).

Recently, we found that WIF1 was strongly expressed in heart from mice at embryonic 16.5–7 days of age, whereas their expression exhibited down-regulation at 14 days and expression continued to decrease thereafter with age. Moreoever, the expression of WIF1 increased in the heart tissue of cTnT^R141W^ transgenic mice, while decreased in the cTnT^R92Q^ transgenic mice.

These results suggest that WIF1 may be involved in the development of the heart and may have important regulation on the pathogenesis of cardiomyopathy.

However, potential effects of WIF1 on heart development, especially on cardiomyopathy have not yet been approached by either in vivo or in vitro studies. Herein we present our investigations, using α-MHC-WIF1 transgenic mice, as well as corresponding cell line, of the effects of WIF1 on heart geometry and function and its probable mechanisms which could be important in documenting underlying regulation of Wnt signaling and Wnt antagonists in heart development and heart diseases.

## Materials and methods

### Animals

The α-MHC-cTnT^R141W^ and α-MHC-cTnT^R92Q^ transgenic mice produced for the present study exhibited phenotypic characteristics consistent with those presented in previous reports (Juan et al. 2008; Tardiff et al. 1999). Genotyping of the transgenic mice was facilitated by the polymerase chain reaction (PCR; cTnT forward, 5′GAACAGGAGGAAGGCTGAGGATGAG and reverse, 5′TATTTCCAGCGCCCGGTGACTTTAG).

The cDNAs encoding human WIF1 (Genbank accession no. NM_007191) were cloned into an expression plasmid under the α-MHC promoter. The construct was microinjected into male pronuclei of fertilized mouse oocytes and implanted into pseudo-pregnant females to generate the transgenic mouse lines. Genotyping was performed by PCR analysis of genomic DNA and the expression of the target gene was analyzed by western blot analysis using antibodie to WIF1 (R&D). Genotyping of transgenic mice was facilitated by the PCR (forward, 5′AGGCATCAGTTGTTCAAGTTGGTT and reverse, 5′ GCAGTTTGCTTTGTCACAGTTCAC) under standard conditions. The WIF1 transgenic mice were maintained on a C57BL/6 J genetic background. All the mice were bred in an AAALAC-accredited facility and the use of animals was approved by the Animal Care and Use Committees of The Institute of Laboratory Animal Science of Peking Union Medical College (GC08-2001).

### Mouse echocardiography

M-mode echocardiography was performed at 1, 3, 6 and 10 months of age on each transgenic mouse with the small animal echocardiography analysis system (Vevo770, Canada) as previously described (Dan et al. 2010; Lu et al. 2012). Briefly, mice were lightly anesthetized by intraperitoneal injection of tribromoethanol at a dose of 180 ml/kg body weight. M-mode echocardiography of the left ventricle was recorded at the tip of the mitral valve apparatus with a 30 MHz transducer. LVAW, LVPW, LVID and EF % were measured. The FS % was calculated as (LVIDD—LVISD)/LVIDD.

### Histological analysis

For light microscopy, cardiac tissue from mice at 6 months of age was fixed in 4 % formaldehyde and mounted in paraffin blocks, and sections were stained with H&E or Masson trichrome as previously described (Javadpour et al. 2003). For transmission electron microscopy (TEM), cardiac tissues were routinely fixed in 2.5 % glutaraldehyde in 0.1 M phosphate buffer (pH 7.4) and postfixed in buffered 1 % osmium tetroxide for 1 h. Samples were then dehydrated using a series of ethanol immersions and embedded in Epon 812. Thin sections were stained with uranyl acetate and lead citrate and examined under a JEM-1230 TEM (Dan et al. 2010; Lu et al. 2012). Myocytes were analysed by an observer blinded to the genotype of the mice.

### RNA extraction, quantification, and reverse-transcription (RT)-PCR

Total RNA was isolated from the tissue or the cell line using TRIzol Reagent (Invitrogen). First-strand cDNA was synthesized from 2 μg of total RNA using random hexamer primers according to the Superscript III reverse transcriptase manufacturer’s protocol (Invitrogen). Detection of mRNA for (nppb), skeletal muscle (actα1) and procollagen type III α1 (Col3α1) were carried out by the RT-PCR using GADPH as normalization under standard conditions (For Nppb forward, 5′TAGCCAGTCTCCAGAACAA and reverse, 5′AACAACCTCAGCCCGTCA; for Acta1 forward, 5′GGCGACTGGAGTCAATAC and reverse, 5′CTCTACAAGCCCGTCATAC; for Col3α1 forward, 5′ CTCAAGAGCGGAGAATACTGG and reverse, 5′CAATGTCATAGGGTGCGATA; for GAPDH forward, 5′CAAGGTCATCCATGACAACTTTG and reverse 5′GTCCACCACCCTGTTGCTGTAG).

### Protein extraction and immunoblotting

Total protein lysates from heart tissue of each transgenic mouse aged 6 months, as well as of cells, were prepared as previously described (Lu et al. 2012). The cytosolic and nuclear fractions were derived following the Nuclear/Cytosol Fractionation Kit manufacturer’s protocol (BioVision). After performing SDS-PAGE and transfer to nitrocellulose (Millipore), the membranes were incubated overnight with antibody to WIF1 (Santa Cruz), β-catenin (Abcam) or c-Myc (Cell Signaling). After incubation with the appropriate secondary antibody for 1 h at room temperature, antibody binding was detected with a HRP-conjugated immunoglobulin G (Santa Crutz) using a chemiluminescent detection system (Westernblotting luminal reagent, Santa Cruz). Bands intersities were quantified using the ImageJ software, and GAPDH was used for normalisation.

### Cell culture

Cells of the rat embryonic ventricular myocyte cell line H9c2 were grown in high glucose DMEM supplemented with 10 % defined fetal bovine serum (HyClone), 100 U/ml penicillin and 100 g/ml streptomycin (HyClone) in a humidified 5 % CO_2_ incubator at 37 °C. The expression construct for WIF1 were generated by cloning full-length human WIF1 cDNA fragment into the pCDNA3.1 (+) vector (Invitrogen). The presence of insert was verified using restriction digestion and DNA sequencing. H9c2 cells were transfected with the constructs using Lipofectamine 2000 (Invitrogen). Stable cell line was established by subsequent selection with 800 μg/ml G418 (Amresco). The cell line was treated for 6 h with LiCl (Sigma) at a final concentration of 50 mM, or Fz-8/Fc (R&D) at a final concentration of 500 ng/ml for 3 h, then cells were harvested for RT-PCR and western bolt analysis.

### Statistical analysis

All measurement data are expressed as mean ± SEM. The statistical significance of differences between groups was analyzed by Student’s *t* test. Differences were considered significant at a *P* value <0.05.

## Results

### Detection of the expression of WIF1

**Fig. 1:**
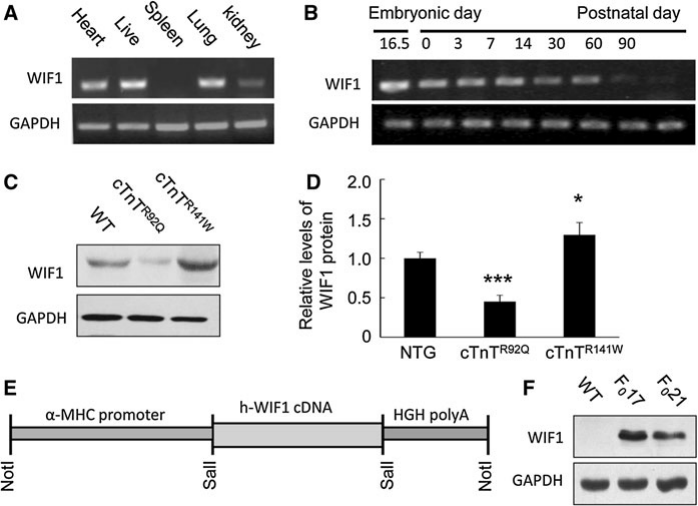
Detection the expression of WIF1 and generation of the transgenic mice (**a**) The expression of WIF1 mRNA in the tissues of neonatal heart, liver, spleen, lung and kidney of WT mice was detected by RT-PCR. **b** Heart tissues were sampled respectively from mice of embryonic 16.5 days, at birth, and at 3, 7, 14, 30, 60 and 90 days of age, and the expression of WIF1 mRNA was detected by RT-PCR. **c** The protein translational level of WIF1 in the cardiomyopathologic hearts from cTnT^R92Q^ and cTnT^R141W^ mice was detected by Western blot. **d** The quantitative analysis of the expression levels of WIF1 using GADPH as normalization. **e** The WIF1 transgenic constructs was generated by inserting the target genes under the control of the α-MHC cardiac-specific promoter and the transgenic mice were created following microinjection. **f** The mouse lines with WIF1 over-expression was selected by the western blot procedure using GADPH as normalization.*P < 0.05; ***P < 0.001 versus NTG mice

Heart, liver, spleen, lung and kidney tissues from wild-type (WT) newborn mice were sampled and the expression of WIF1 mRNA was detected by the RT-PCR method. WIF1 showed high level of expression in heart tissue (Fig. 1a). Heart tissues were sampled respectively from WT mice at embryonic 16.5 days, day 0, and at 3, 7, 14, 30, 60 and 90 days of age, and the expression of WIF1 was detected. WIF1 was strongly expressed in heart from mice at embryonic 16.5–7 days of age, whereas their expression exhibited down-regulation at 14 days and expression continued to decrease thereafter with age (Fig. 1b).

The expression of mutant cTnT^R92Q^ and cTnT^R141W^ in the mouse heart evoked contrasting pathologic phenotypes, evinced as HCM and DCM. The expression of WIF1 was detected by western blot in the cTnT^R92Q^ and cTnT^R141W^ heart. WIF1 was down-regulated in the cTnT^R92Q^ heart (*P* < 0.001), but was up-regulated in the cTnT^R141W^ (*P* < 0.05; Fig. 1c, d). The results suggest that WIF1 are involved in the development of the heart and may have contrasting different effects on the pathogenesis of cardiomyopathy.

### Generation and identification of the WIF1 transgenic mice

To study the modulation of WIF1 on heart geometry and function and its probable mechanisms, C57BL/6 J mice carrying the WIF1 gene was established (Fig. 1e). Two lines of WIF1 transgenic mice, founder 17 and 21, with high levels of expression were selected from among 36 founders by the western blot procedure (Fig. 1f).

The WIF1 transgenic mice were indistinguishable from their NTG littermates at birth and in youth. No death was observed in the NTG transgenic mice, while the extent of mortality in WIF1 mice was around 4 % (1 of 25) within 10 months (*P* = 0.327 versus NTG mice).

### WIF1 caused dysfunction of heart in transgenic mice

Ventricular size and function of the two transgenic lines were assessed using echocardiography. Dates from founder 21 given the similar tendency of phentype to founder 17 for WIF1 mice at 6 months of age (Supplemental Table S1).

**Fig. 2:**
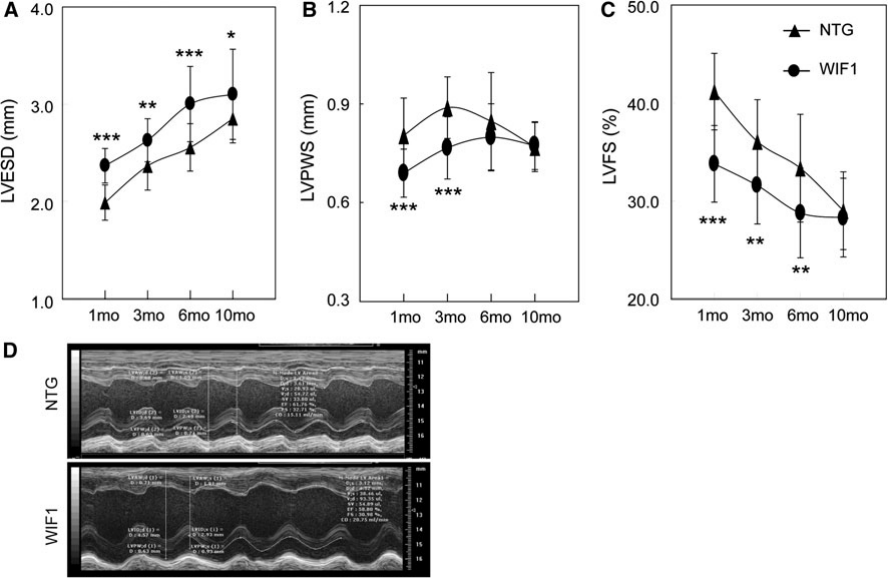
The effects of WIF1 on heart dimensions and function in transgenic mice.Echocardiographic parameters of LV end-systole diameter (LVESD) (**a**), LV posterior wall at end-systole (LVPWS) (**b**) and LV fractional shortening (LVFS) (**c**) were compared with NTG mice at 1, 3, 6 and 10 months of age. **d** Representative M-mode echocardiographic images of the left ventricle long-axis from 6 months old NTG and transgenic mice. *P < 0.05; **P < 0.01; ***P < 0.001 versus NTG mice at the same month of age

The deterioration was demonstrated by the 11.2 % increased left ventricular diameter at end-systole (LVESD) (*P* < 0.01, WIF1 mice versus NTG mice), 13.8 % reduced left ventricular posterior wall thickness at end-systole (LVPWS) (*P* < 0.001, WIF1 mice versus NTG mice), and 12.5 % reduced left ventricular percent fractional shortening (LVFS) (*P* < 0.01, WIF1 mice versus NTG mice) in mice at 3 months of age (Fig. 2a–c).

The representative M-mode echocardiograms from founder 17 at the age of 6 months old were shown in Fig. 2d. The other parameters of M-mode echocardiography from the NTG and WIF1 transgenic mice at 1, 3, 6 and 10 months of ages were summarized in supplemental table S2 (Founder 17 of WIF1 transgenic mice).

To sum up, the the overexprssion of WIF1 in heart exhibited thin-walled and dilated left and right ventricles when compared with NTG hearts. The WIF1 transgenic mice developed a progressive LV dilation and dysfunction associated with a progressive decrease of contractile function, evidenced by decreased LV percent fractional shortening (FS %) which exhibited a significance from 1 months of age compared with NTG mice.

### Morphological changes of myocytes in the WIF1 transgenic mice

**Fig. 3:**
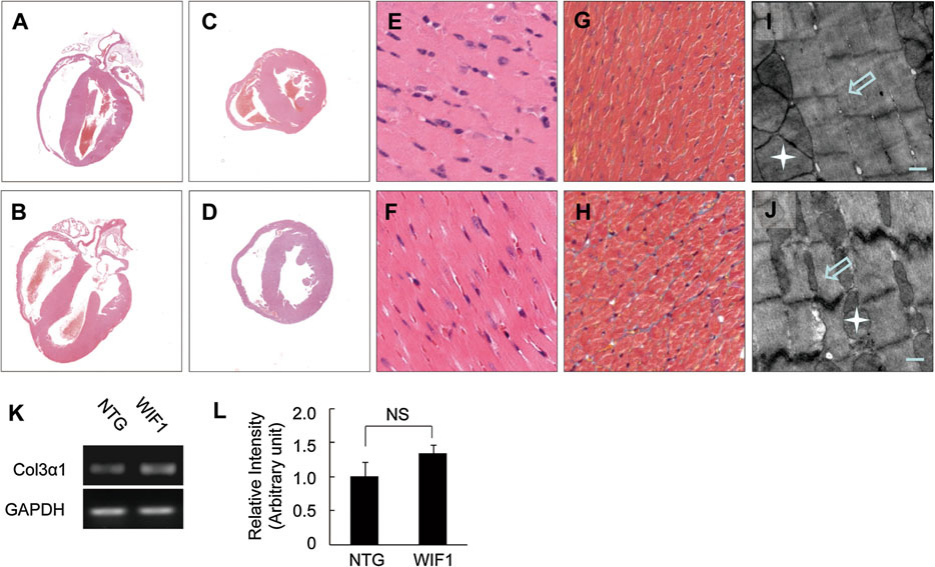
Profile of histopathological and ultrastructural in hearts of transgenic mice at 6 months of age. **a**–**d** Shown are H&E staining patterns of whole-heart longitudinal sections and cross-sections from 6 months old NTG (**a** and **c**) and transgenic mice (**b** and **d**) (magnification, ×20). **e**–**f** Shown are H&E stained sections of *left ventricle*, showing disparate in pathological changes from 6 months old NTG (**e**) and transgenic mice (**f**) (magnification ×400). **g**–**h** Masson trichrome staining of sections of *left ventricle* from 6 months old NTG (**g**) and transgenic mice (**h**);myocytes, stained red, collagenous tissue, stained green (magnification ×400). **i**–**j** TEM showing *left ventricular* free walls from 6 months old NTG (**i**) and transgenic mice (**j**) [*white scale bars* = 0.5 μm, sarcomeres (*white hollow arrow*) and mitochondria (*white star*)]. **k** The expression of Col3α1 was detected by RT-PCR. **l** The quantitative analysis of the expression of Col3α1 using GAPDH for normalisation (NS, no significance)

By light microscopy (Fig. 3a–h), no significant changes were observed in the heart tissues of WIF1 transgenic mice, except for occurrence of myocyte disarray in the heart tissues of WIF1 transgenic mice (Fig. 3e, f). Masson trichrome staining indicated that a small proportion of collagen accumulated in the interstitial space in the WIF1 transgenic mice (Fig. 3g, h).

Ultrastructural characteristics of heart tissues are summarized in Fig. 3i, j. Slightly elongated myofrils with swollen sarcoplasmic reticulum were observed in WIF1 mice.

Expression level of Col3α1 mRNA were slightly increased in WIF1 mice compared with NTG mice (*P* = 0.0754, WIF1 mice versus NTG mice; Fig. 3k, l), consistent with the result of Masson trichrome staining (Fig. 3g, h).

### WIF1 inhibited β-catenin pathway hypertrophic markers in heart tissues of the transgenic mice

To assess whether functional Wnt signaling is inhibited in the heart tissues of the transgenic mice, we examined by use of the western blot procedure the occurrence of cytosolic and nuclear β-catenin, the crux of the canonical Wnt signal pathway.

**Fig. 4:**
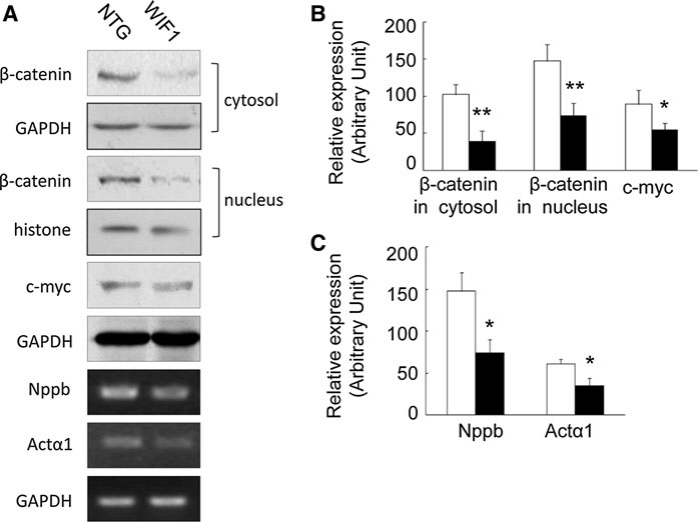
The effects of WIF1 on β-catenin pathway and hypertrophic markersin the hearts at 6 months of age. **a** Immunblots of subcelluar protein extracts were probed using respective antobodies in the western blot procedure. **b** The quantitative analysis of the expression levels of proteins using GADPH as normalization

Accumulation of β-catenin in nucleus and cytoplasm were decreased in WIF1 mice (Fig. 4a, b, *P* < 0.01, WIF1 mice versus NTG mice), concomitant with decreased expression of transcription factors c-myc (Fig. 4a, b, *P* < 0.05, WIF1 mice versus NTG mice), coding sequences for which are target gene for β-catenin, consistent with what is known about the β-catenin/TCF pathway (Moon et al. 2002; Nakamura et al. 2003).

The expression of (nppb; Fig. 4a, b, *P* < 0.05) and skeletal muscle actin α1 (actα1; Fig. 4a, b, *P* < 0.05) mRNA (both of which were commonly used as hypertrophic markers) was decreased in the WIF1 mice compared with the corresponding expression levels in NTG mice.

### WIF1 blocks hypertrophic markers through inhibition of the β-catenin pathway in the H9c2 Cell Line

**Fig. 5:**
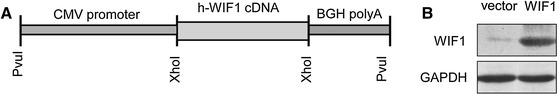
The establishment of WIF1 overexpression cell line. **a** The plasmids for stable expression of WIF1 was constructed. **b** Stable cell lines were selected by Western blot with respective antibodies for WIF1. GAPDH was used as normalization

To assess the effect of WIF1 in vitro, H9c2 cells were transfected with construct incorporating WIF1 (Fig. 5a). Stable cell line overexpressing target gene were identified by the western blot procedure (Fig. 5b).

**Fig. 6:**
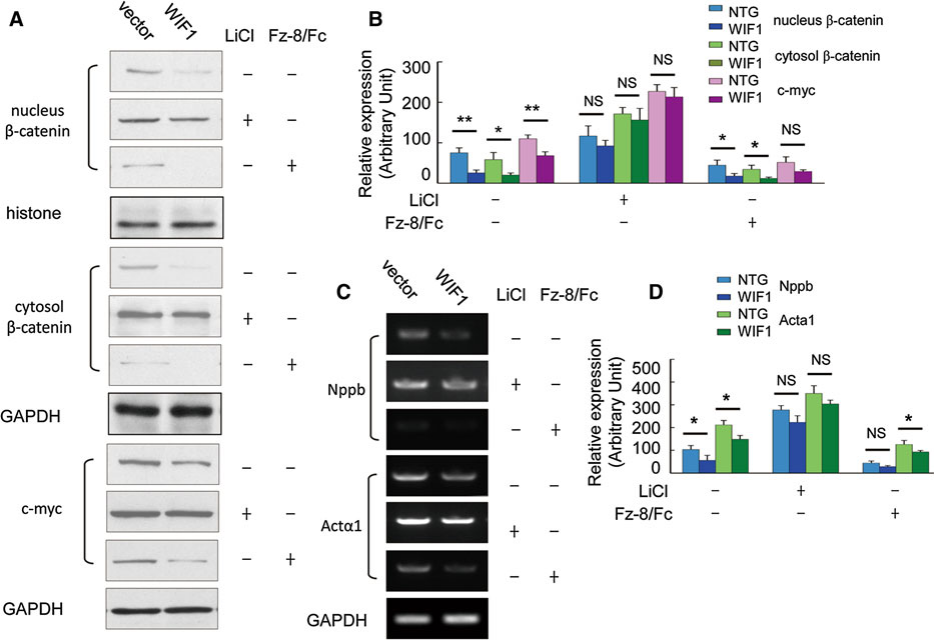
WIF1 blocks hypertrophic markers through inhibition of the β-catenin pathway in the H9c2 cell Line. **a** Stable cell lines expressing the vector with no insert, or WIF1 cDNA were treated with LiCl or Fz-8/Fc, and subcelluar protein extracts were analyzed using antibody to β-catenin, and c-myc was also dectected in total lysates by Western blot. **b** The quantitative analysis of the expression levels of proteins using GADPH as normalization. **c** Transcripts for nppb andactα1 were detected by RT-PCR method from stable cell lines expressing the vector with no insert, or WIF1 cDNA treated with LiCl or Fz-8/Fc. **d** The quantitative analysis of the expression levels of proteins using GADPH as normalization

Accumulation of β-catenin in nucleus (Fig. 6a, b, *P* < 0.01, WIF1 cell versus vector cell) and cytoplasm (Fig. 6a, b, *P* < 0.05, WIF1 cell versus vector cell) were decreased in WIF1 cell line, concomitant with decreased expression of c-myc (Fig. 6a, b, *P* < 0.01, WIF1 cell versus vector cell). The expression of nppb (Fig. 6c, d, *P* < 0.05) and actα1 (Fig. 6c and D, *P* < 0.05) mRNA were decreased in the WIF1 cell compared with the corresponding expression levels in vector cell.

LiCl can bind with and inhibit GSK-3*β* and, hence, activate Wnt signaling selectively via the β-catenin/TCF pathway. Frizzled-8/Fc chimeric protein (Fz-8/Fc), an antagonist for Wnt8A, and potentially for other Wnts, decreases the expression of β-catenin to its basal level (Moon et al. 2002; Nakamura et al. 2003).

To test whether the presence of LiCl or Fz-8/Fc could worsen or reverse the effect of WIF1 on the expression of the hypertrophic markers, as has been observed in vivo (Fig. 4), the cells (stable H9c2 cell line overexpressing WIF1) were treated with LiCl or Fz-8/Fc, and the expression of β-catenin, c-myc, nppb and actα1 were determined (Fig. 6). Increase in occurrence of β-catenin in nuclear (Fig. 6a, b, no significance (NS), WIF1 cell versus vector cell) and cytoplasmic (Fig. 6a, b, NS, WIF1 cell versus vector cell) protein subfractions, as well as increase of c-myc (Fig. 6a, b, NS, WIF1 cell versus vector cell) in the WIF1 cells treated with LiCl, is consistent with observable increase in expression of nppb (Fig. 6c, d, NS, WIF1 cell versus vector cell) and actα1 (Fig. 6c, d, NS, WIF1 cell versus vector cell) mRNAs, while the occurrence of β-catenin in nuclear (Fig. 6a, b, *P* < 0.05, WIF1 cell versus vector cell) and cytoplasmic (Fig. 6a, b, *P* < 0.05, WIF1 cell versus vector cell) protein subfractions were further inhibited as well as decreased of c-myc (Fig. 6a, b, NS, WIF1 cell versus vector cell) in the WIF1 cells treated with Fz-8/Fc, in consistent with observable decrease in the expression of nppb (Fig. 6c, d, NS, WIF1 cell versus vector cell) and actα1 (Fig. 6c, d, *P* < 0.05, WIF1 cell versus vector cell) mRNAs.

## Discussion

The Wnt signaling pathway is a major regulator of cell proliferation, migration and differentiation, controlling embryogenesis, adult tissue homeostasis and tumor progression (MacDonald et al. 2009). WIF1 is a secreted protein that binds to Wnt proteins and inhibits their activities (Hsieh et al. 1999), and it can directly bind to Wnt proteins and inhibit Wnts from binding to their receptors in vertebrates (Hsieh et al. 1999). WIF1 inhibits rod production in retinal histogenesis in the mouse by binding to Wnt4 (Hunter et al. 2004). Most of the published reports have indicated that WIF1 is a frequent target of epigenetic inactivation in cancers (Ding et al. 2009; Urakami et al. 2006; Mazieres et al. 2004; Ai et al. 2006; Clément et al. 2008; Chan et al. 2007; Taniguchi et al. 2005; Lin et al. 2007, 2008; Kim et al. 2007). The expression of WIF1 is first detectable at the start of somitogenesis in the paraxial mesoderm during embryogenesis in *Xenopus* and zebrafish. The WIF1 expression continues in adults in the heart, lungs and cartilage-mesenchyme interfaces of various species (Surmann-Schmitt et al. 2009), and it is indicated that WIF1 enhances cardiomyogenesis (Buermans et al. 2010).

It is indicated that WIF1 mRNA can be detected as early as the developmental stage E11, and expression persists to adulthood, and after birth, the expression level of WIF1 decreased in the cortex and diencephalon (Hu et al. 2008). In our present study, we find prominent level of WIF1 in the heart of mice at embryonic 16.5–7 days of age (Fig. 1b). Furthermore, the expression of WIF1 was found to be regulated in the heart of transgenic mice model of cardiomyopathy (Fig. 1c, d).

To study the effect of WIF1 on heart geometry and function, we generated WIF1 transgenic mice with genetic material for this component specifically expressed in heart tissue (Figs. 2 and 3). In the present work, the most noteworthy findings were that the overexprssion of WIF1 in heart exhibited thin-walled and dilated left and right ventricles when compared with NTG hearts, and the WIF1 transgenic mice developed a progressive LV dilation and dysfunction associated with a progressive decrease of contractile function, evidenced by decreased LVFS which exhibited a significance from 1 months of age compared with NTG mice. Moreover, the presence of WIF1 in the heart caused swollen sarcoplasmic reticulum and elongated myofrils in heart tissue. Overall, WIF1 showed a similar phenotype to the cTnT^R141W^ transgenic mice of cardiomyopathy (Juan et al. 2008; Lu et al. 2012).

The importance of nppb as a diagnostic and therapeutic modality in cardiovascular disease is well known, it also acts as a local regulator of ventricular remodeling and a modifier of cardiac gene expression (Lanfear et al. 2007; Tamura et al. 2000; Tsybouleva et al. 2004). Nppb is a cardiac hormone produced primarily by ventricular myocytes, and its plasma concentrations are markedly elevated in patients with congestive heart failure and acute myocardial infarction. Our results indicate that WIF1 significantly decreases the expression levels of nppb, suggesting that WIF1 regulate cardiac hypertrophy and that remodeling is in part due to interaction with nppb.

Actα1 is present in the developing heart and it constitutes up to 20 % of the striated actin of the adult heart. Mice lacking skeletal actin died in the early neonatal period (Crawford et al. 2002). Our results show that levels of actα1 mRNA are significantly decreased in the WIF1 mice. Since actα1 is a multifunctional protein that interacts with many proteins involved in folding, polymerisation, contractility and regulation of contractility (Feng and Marston 2009), decreased levels may affect any of those functions, suggesting that actα1 could be involved in mechanisms by which WIF1 leads to development of heart dysfunction.

The Wnt/β-catenin pathway, which appears to antagonize cardiomyocyte differentiation and/or to restrict the size of the cardiogenic field, is critical for endocardial cushion morphogenesis, outflow tract development, and valve formation in later development (Hurlstone et al. 2003). Specific Wnt ligands bind to their target membrane receptors and interfere with the multi-protein destruction complex, resulting in downstream activation of gene transcription by β-catenin. Unphosphorylated β-catenin accumulates and associates with nuclear transcription factors, leading to the eventual transcription and expression of target genes such as c-myc (Park et al. 2005).

Our results indicate that WIF1 decreases, the accumulation of β-catenin in the nucleus and cytoplasm, consistent with the expression of c-myc in the WIF1 transgenic mice (Fig. 4). In allied experiments, the similar results were found in the cell lines over-expressing WIF1 (Fig. 6).

LiCl can activate Wnt signaling selectively via the β-catenin/TCF pathway (Moon et al. 2002). Fz-8/Fc, an antagonist for Wnt8A and potentially for other Wnts, was shown to decrease β-catenin down to the basal level (Nakamura et al. 2003). We found that WIF1 expression alone in WIF1 mice or in H9c2 cells decreases the accumulation of β-catenin in cytoplasm and the nucleus, consistent with a decrease in c-myc expression as well as nppb and actα1 expression. Furthermore, the inhibition of the Wnt signal induced by WIF1 could be reversed by LiCl, further suggesting that WIF1 acts as a negative regulator of Wnt pathway in the myocyte and is involved in the pathological development of heart diseases.

In summary, we have found that expression of WIF1 is altered in the cardiomyopathy mouse model and that WIF1 was shown to have harmful effects on function and structure of hearts. The effects on β-catenin pathway maybe the course of the former. It is anticipated that our findings will contribute to expansion our understanding of WIF1 biological function on heart development and possible therapeutic strategy to control heart diseases.

### Electronic supplementary material

Below is the link to the electronic supplementary material.

Supplementary material (DOCX 20 kb)

## References

[CR1] Ai L, Tao Q, Zhong S, Fields CR, Kim WJ, Lee MW, Cui Y, Brown KD, Robertson KD (2006) Inactivation of Wnt inhibitory factor-1 (WIF1) expression by epigenetic silencing is a common event in breast cancer. Carcinogenesis 27:1341–1348 16501252 10.1093/carcin/bgi379

[CR2] Buermans HP, van Wijk B, Hulsker MA, Smit NC, den Dunnen JT, van Ommen GB, Moorman AF, van den Hoff MJ, ‘t Hoen PA (2010) Comprehensive gene-expression survey identifies wif1 as a modulator of cardiomyocyte differentiation. PLoS ONE 5:e15504 21179454 10.1371/journal.pone.0015504 PMC3001492

[CR3] Chan SL, Cui Y, van Hasselt A, Li H, Srivastava G, Jin H, Ng KM, Wang Y, Lee KY, Tsao GS, Zhong S, Robertson KD, Rha SY, Chan AT, Tao Q (2007) The tumor suppressor Wnt inhibitory factor 1 is frequently methylated in nasopharyngeal and esophageal carcinomas. Lab Invest 87:644–650 17384664 10.1038/labinvest.3700547

[CR4] Clément G, Guilleret I, He B, Yagui-Beltrán A, Lin YC, You L, Xu Z, Shi Y, Okamoto J, Benhattar J, Jablons D (2008) Epigenetic alteration of the Wnt inhibitory factor-1 promoter occurs early in the carcinogenesis of Barrett’s esophagus. Cancer Sci 99:46–53 18005197 10.1111/j.1349-7006.2007.00663.x PMC11158554

[CR5] Cohen ED, Tian Y, Morrisey EE (2008) Wnt signaling: an essential regulator of cardiovascular differentiation, morphogenesis and progenitor self-renewal. Development 135:789–798 18263841 10.1242/dev.016865

[CR6] Crawford K, Flick R, Close L, Shelly D, Paul R, Bove K, Kumar A, Lessard J (2002) Mice lacking skeletal muscle actin show reduced muscle strength and growth deficits and die during the neonatal period. Mol Cell Biol 22:5887–5896 12138199 10.1128/MCB.22.16.5887-5896.2002 PMC133984

[CR7] Dan L, Hong L, Xiaojuan Z, Haitao S, Lan H, Chuan Q, Lianfeng Z (2010) LMNA E82 K mutation activates FAS and mitochondrial pathways of apoptosis in heart tissue specific transgenic mice. PLoS ONE 5:e15167 21151901 10.1371/journal.pone.0015167 PMC2997782

[CR8] Ding Z, Qian YB, Zhu LX, Xiong QR (2009) Promoter methylation and mRNA expression of DKK-3 and WIF-1 in hepatocellular carcinoma. World J Gastroenterol 15:2595–2601 19496188 10.3748/wjg.15.2595 PMC2691489

[CR9] Feng JJ, Marston S (2009) Genotype-phenotype correlations in ACTA1 mutations that cause congenital myopathies. Neuromuscul Disord 19:6–16 18976909 10.1016/j.nmd.2008.09.005

[CR10] Hsieh JC, Kodjabachian L, Rebbert ML, Rattner A, Smallwood PM, Samos CH, Nusse R, Dawid IB, Nathans J (1999) A new secreted protein that binds to Wnt proteins and inhibits their activities. Nature 398:431–436 10201374 10.1038/18899

[CR11] Hu YA, Zhao CJ (2010) Research progress of Wif1 in development of nervous system. Zhejiang Da Xue Xue Bao Yi Xue Ban 39:93–96 20175243 10.3785/j.issn.1008-9292.2010.01.016

[CR12] Hu YA, Gu X, Liu J, Yang Y, Yan Y, Zhao C (2008) Expression pattern of Wnt inhibitor factor 1 (Wif1) during the development in mouse CNS. Gene Expr Patterns 8:515–522 18586116 10.1016/j.gep.2008.06.001

[CR13] Hunter DD, Zhang M, Ferguson JW, Koch M, Brunken WJ (2004) The extracellular matrix component WIF-1 is expressed during, and can modulate, retinal development. Mol Cell Neurosci 27:477–488 15555925 10.1016/j.mcn.2004.08.003 PMC2935895

[CR14] Hurlstone AF, Haramis AP, Wienholds E, Begthel H, Korving J, Van Eeden F, Cuppen E, Zivkovic D, Plasterk RH, Clevers H (2003) The Wnt/beta-catenin pathway regulates cardiac valve formation. Nature 425:633–637 14534590 10.1038/nature02028

[CR15] Javadpour MM, Tardiff JC, Pinz I, Ingwall JS (2003) Decreased energetics in murine hearts bearing the R92Q mutation in cardiac troponin T. J Clin Invest 112:768–775 12952925 10.1172/JCI15967 PMC182182

[CR16] Juan F, Wei D, Xiongzhi Q, Ran D, Chunmei M, Lan H, Chuan Q, Lianfeng Z (2008) The changes of the cardiac structure and function in cTnTR141 W transgenic mice. Int J Cardiol 128:83–90 18606313 10.1016/j.ijcard.2008.03.006

[CR17] Kawano Y, Kypta R (2003) Secreted antagonists of the Wnt signalling pathway. J Cell Sci 116:2627–2634 12775774 10.1242/jcs.00623

[CR18] Kim J, You L, Xu Z, Kuchenbecker K, Raz D, He B, Jablons D (2007) Wnt inhibitory factor inhibits lung cancer cell growth. J Thorac Cardiovasc Surg 133:733–737 17320573 10.1016/j.jtcvs.2006.09.053

[CR19] Lanfear DE, Stolker JM, Marsh S, Rich MW, McLeod HL (2007) Genetic variation in the B-type natiuretic peptide pathway affects BNP levels. Cardiovasc Drugs Ther 21:55–62 17340039 10.1007/s10557-007-6007-5

[CR20] Lin YC, You L, Xu Z, He B, Yang CT, Chen JK, Mikami I, Clément G, Shi Y, Kuchenbecker K, Okamoto J, Kashani-Sabet M, Jablons DM (2007) Wnt inhibitory factor-1 gene transfer inhibits melanoma cell growth. Hum Gene Ther 18:379–386 17472570 10.1089/hum.2006.005

[CR21] Liu J, Lam JB, Chow KH, Xu A, Lam KS, Moon RT, Wang Y (2008) Adiponectin stimulates Wnt inhibitory factor-1 expression through epigenetic regulations involving the transcription factor specificity protein 1. Carcinogenesis 29:2195–2202 18701434 10.1093/carcin/bgn194

[CR22] Lu D, Ma Y, Zhang W, Bao D, Dong W, Lian H, Huang L, Zhang L (2012) Knockdown of cytochrome P450 2E1 inhibits oxidative stress and apoptosis in the cTnTR141 W dilated cardiomyopathy transgenic mice. Hypertension 60:81–89 22665122 10.1161/HYPERTENSIONAHA.112.191478

[CR23] Macdonald BT, Semenov MV, He X (2007) SnapShot: Wnt/beta-catenin signaling. Cell 131:1204 18083108 10.1016/j.cell.2007.11.036

[CR24] MacDonald BT, Tamai K, He X (2009) Wnt/beta-catenin signaling: components, mechanisms, and diseases. Dev Cell 17:9–26 19619488 10.1016/j.devcel.2009.06.016 PMC2861485

[CR25] Malinauskas T, Aricescu AR, Lu W, Siebold C, Jones EY (2011) Modular mechanism of Wnt signaling inhibition by Wnt inhibitory factor 1. Nat Struct Mol Biol 18:886–893 21743455 10.1038/nsmb.2081 PMC3430870

[CR26] Mazieres J, He B, You L, Xu Z, Lee AY, Mikami I, Reguart N, Rosell R, McCormick F, Jablons DM (2004) Wnt inhibitory factor-1 is silenced by promoter hypermethylation in human lung cancer. Cancer Res 64:4717–4720 15256437 10.1158/0008-5472.CAN-04-1389

[CR27] Moon RT, Bowerman B, Boutros M, Perrimon N (2002) The promise and perils of Wnt signaling through beta-catenin. Science 296:1644–1646 12040179 10.1126/science.1071549

[CR28] Nakamura T, Sano M, Songyang Z, Schneider MD (2003) A Wnt- and beta -catenin-dependent pathway for mammalian cardiac myogenesis. Proc Natl Acad Sci 100:5834–5839 12719544 10.1073/pnas.0935626100 PMC156287

[CR29] Nakaya N, Lee HS, Takada Y, Tzchori I, Tomarev SI (2008) Zebrafish olfactomedin 1 regulates retinal axon elongation in vivo and is a modulator of Wnt signaling pathway. J Neurosci 28:7900–7910 18667622 10.1523/JNEUROSCI.0617-08.2008 PMC2692209

[CR30] Park JI, Kim SW, Lyons JP, Ji H, Nguyen TT, Cho K, Barton MC, Deroo T, Vleminckx K, Moon RT, McCrea PD (2005) Kaiso/p120-catenin and TCF/beta-catenin complexes coordinately regulate canonical Wnt gene targets. Dev Cell 8:843–854 15935774 10.1016/j.devcel.2005.04.010

[CR31] Semenov MV, Habas R, Macdonald BT, He X (2007) SnapShot: noncanonical Wnt signaling pathways. Cell 131:1378 18160045 10.1016/j.cell.2007.12.011

[CR32] Surmann-Schmitt C, Widmann N, Dietz U, Saeger B, Eitzinger N, Nakamura Y, Rattel M, Latham R, Hartmann C, von der Mark H, Schett G, von der Mark K, Stock M (2009) Wif-1 is expressed at cartilage-mesenchyme interfaces and impedes Wnt3a-mediated inhibition of chondrogenesis. J Cell Sci 122:3627–3637 19755491 10.1242/jcs.048926

[CR33] Tamura N, Ogawa Y, Chusho H, Nakamura K, Nakao K, Suda M, Kasahara M, Hashimoto R, Katsuura G, Mukoyama M, Itoh H, Saito Y, Tanaka I, Otani H, Katsuki M (2000) Cardiac fibrosis in mice lacking brain natriuretic peptide. Proc Natl Acad Sci USA 97:4239–4244 10737768 10.1073/pnas.070371497 PMC18212

[CR34] Tang Y, Simoneau AR, Liao WX, Yi G, Hope C, Liu F, Li S, Xie J, Holcombe RF, Jurnak FA, Mercola D, Hoang BH, Zi X (2009) WIF1, a Wnt pathway inhibitor, regulates SKP2 and c-myc expression leading to G1 arrest and growth inhibition of human invasive urinary bladder cancer cells. Mol Cancer Ther 8:458–468 19174556 10.1158/1535-7163.MCT-08-0885 PMC2768341

[CR35] Taniguchi H, Yamamoto H, Hirata T, Miyamoto N, Oki M, Nosho K, Adachi Y, Endo T, Imai K, Shinomura Y (2005) Frequent epigenetic inactivation of Wnt inhibitory factor-1 in human gastrointestinal cancers. Oncogene 24:7946–7952 16007117 10.1038/sj.onc.1208910

[CR36] Tardiff JC, Hewett TE, Palmer BM, Olsson C, Factor SM, Moore RL, Robbins J, Leinwand LA (1999) Cardiac troponin T mutations result in allele-specific phenotypes in a mouse model for hypertrophic cardiomyopathy. J Clin Invest 104:469–481 10449439 10.1172/JCI6067 PMC408522

[CR37] Thierfelder L, Watkins H, MacRae C, Lamas R, McKenna W, Vosberg HP, Seidman JG, Seidman CE (1994) Alpha-tropomyosin and cardiac troponin T mutations cause familial hypertrophic cardiomyopathy: a disease of the sarcomere. Cell 77:701–712 8205619 10.1016/0092-8674(94)90054-X

[CR38] Tsybouleva N, Zhang L, Chen S, Patel R, Lutucuta S, Nemoto S, DeFreitas G, Entman M, Carabello BA, Roberts R, Marian AJ (2004) Aldosterone, through novel signaling proteins, is a fundamental molecular bridge between the genetic defect and the cardiac phenotype of hypertrophic cardiomyopathy. Circulation 109:1284–1291 14993121 10.1161/01.CIR.0000121426.43044.2B PMC2779533

[CR39] Urakami S, Shiina H, Enokida H, Kawakami T, Tokizane T, Ogishima T, Tanaka Y, Li LC, Ribeiro-Filho LA, Terashima M, Kikuno N, Adachi H, Yoneda T, Kishi H, Shigeno K, Konety BR, Igawa M, Dahiya R (2006) Epigenetic inactivation of Wnt inhibitory factor-1 plays an important role in bladder cancer through aberrant canonical Wnt/β-catenin signaling pathway. Clin Cancer Res 12:383–391 16428476 10.1158/1078-0432.CCR-05-1344

[CR40] Watkins H, McKenna WJ, Thierfelder L, Suk HJ, Anan R, O’Donoghue A, Spirito P, Matsumori A, Moravec CS, Seidman JG et al (1995) Mutations in the genes for cardiac troponin T and alpha-tropomyosin in hypertrophic cardiomyopathy. N Engl J Med 332:1058–1064 7898523 10.1056/NEJM199504203321603

[CR41] Wissmann C, Wild PJ, Kaiser S, Roepcke S, Stoehr R, Woenckhaus M, Kristiansen G, Hsieh JC, Hofstaedter F, Hartmann A, Knuechel R, Rosenthal A, Pilarsky C (2003) WIF1, a component of the Wnt pathway, is down-regulated in prostate, breast, lung, and bladder cancer. J Pathol 201:204–212 14517837 10.1002/path.1449

