# Correction to: WIF1 causes dysfunction of heart in transgenic mice

**DOI:** 10.1007/s11248-024-00403-y

**Published:** 2024-08-15

**Authors:** Dan Lu, Wei Dong, Xu Zhang, Xiongzhi Quan, Dan Bao, Yingdong Lu, Lianfeng Zhang

**Affiliations:** 1 Key Laboratory of Human Disease Comparative Medicine, Ministry of Health, Institute of Laboratory Animal Science, Chinese Academy of Medical Sciences and Comparative Medical Center, Peking Union Medical College, Beijing, People’s Republic of China; 2 Key Laboratory of Human Disease Animal Model, State Administration of Traditional Chinese Medicine, Institute of Laboratory Animal Science, Chinese Academy of Medical Sciences and Comparative Medical Center, Peking Union Medical College, Building 5, Panjiayuan Nanli, Chaoyang District, Beijing, People’s Republic of China

**Correction to: Transgenic Res (2013) 22:1179–1189** 10.1007/s11248-013-9738-z

In the original publication, panels E and F of Fig. 3 were interchanged. The corrected version of Fig. 3 is published here.

**Fig. 3:**
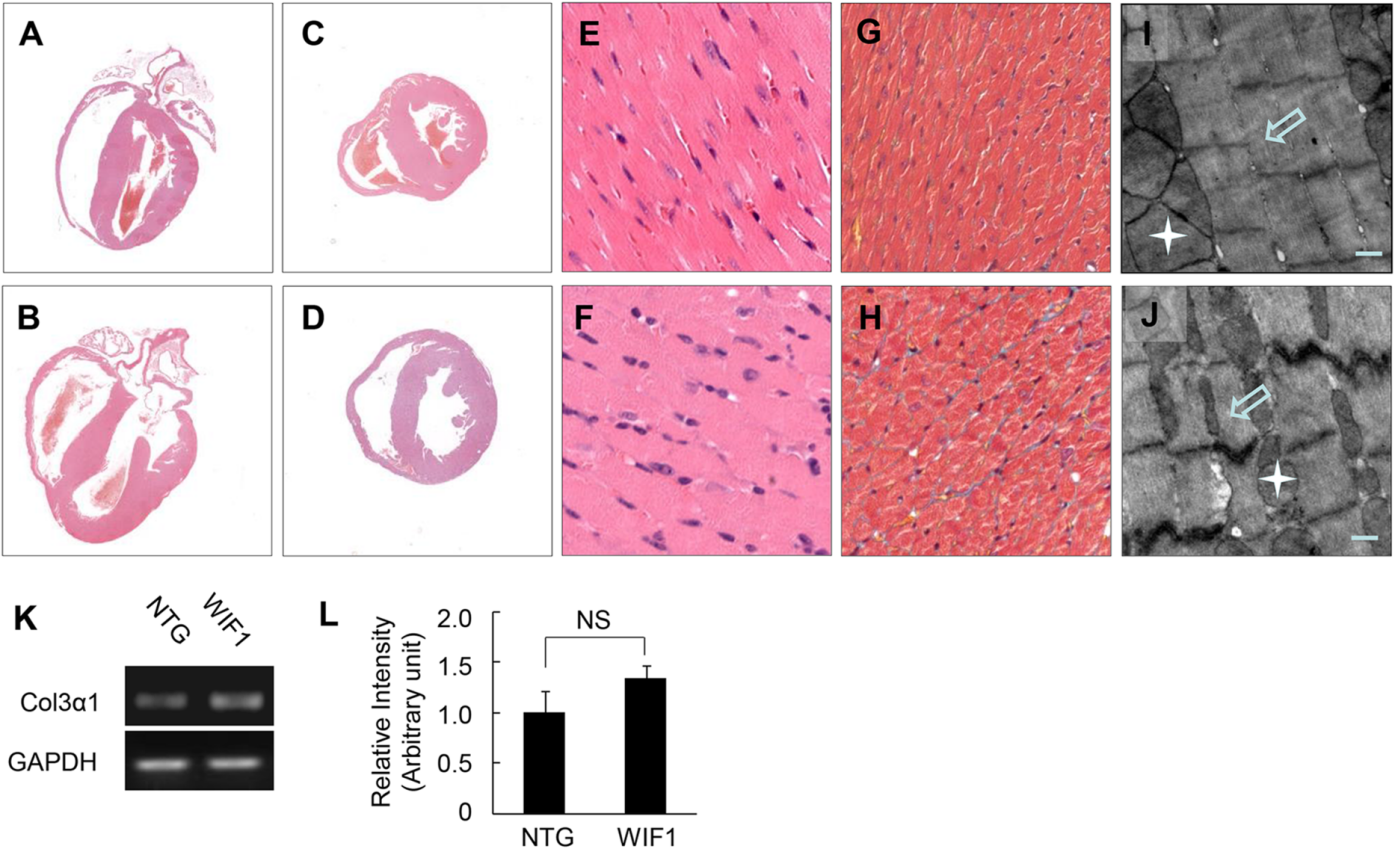
Profile of histopathological and ultrastructural in hearts of transgenic mice at 6 months of age. **a–d** Shown are H&E staining patterns of whole-heart longitudinal sections and cross-sections from 6 months old NTG (**a** and **c**) and transgenic mice (**b** and **d**) (magnification, × 20). **e–f** Shown are H&E stained sections of *left ventricle*, showing disparate in pathological changes from 6 months old NTG (**e**) and transgenic mice (**f**) (magnification × 400). **g–h** Masson trichrome staining of sections of *left ventricle* from 6 months old NTG (**g**) and transgenic mice (**h**);myocytes, stained red, collagenous tissue, stained green (magnification × 400). **i–j** TEM showing *left ventricular* free walls from 6 months old NTG (**i**) and transgenic mice (**j**) [*white scale bars* = 0.5 µm, sarcomeres (*white hollow arrow*) and mitochondria (*white star*)]. **k** The expression of Col3α1 was detected by RT-PCR. **l** The quantitative analysis of the expression of Col3α1 using GAPDH for normalisation (NS, no significance)

