# Supplementary material for: WIF1 causes dysfunction of heart in transgenic mice
Source: Transgenic Res. 2013 Aug 7;22(6):1179–89. doi: 10.1007/s11248-013-9738-z (PMC3835953; doi:10.1007/s11248-013-9738-z)
Supplement: Supplementary file 1 — Supplementary material (DOCX 20 kb) [file 11248_2013_9738_MOESM1_ESM.docx]

Supplemental table S1. Echocardiographic characteristics of WIF1 transgenic mice

(line 17 and line 21) at 6 months of age

| Group  Number | NTG  *n* = 24 | WIF1(line 21)  *n* = 16 | WIF1(line 17)  *n* = 25 |
| --- | --- | --- | --- |
| LVEDD, mm | 3.84±0.21 | 4.23±0.37^***^ | 4.21±0.38^***^ |
| LVESD, mm | 2.56±0.24 | 2.96±0.37^***^ | 3.00±039^***^ |
| LVPWD, mm | 0.58±0.07 | 0.63±0.07 | 0.62±0.08 |
| LVPWS, mm | 0.85±0.15 | 0.81±0.11 | 0.80±0.10 |
| LVAWD, mm | 0.75±0.08 | 0.69±0.08^*^ | 0.70±0.07^*^ |
| LVAWS, mm | 1.11±0.15 | 0.88±0.13^***^ | 0.87±0.10^***^ |
| LVEF, % | 62.20±7.21 | 56.77±6.17^*^ | 55.40±7.19^**^ |
| LVFS, % | 33.35±5.50 | 29.70±3.82^*^ | 28.76±4.58^**^ |
| HR, bpm | 439.47±49.85 | 427.07±33.65 | 428.54±33.94 |

LVEDD: left ventricle (LV) end-diastole diameter; LVESD: LV end-systole diameter; LVPWD: LV posterior wall at end-diastole; LVPWS: LV posterior wall at end-systole; LVAWD: LV anterior wall at end-diastole; LVAWS: LV anterior wall at end-systole; LVEF: LV ejection fraction; LVFS: LV fractional shortening; HR: heart rate. ^*^*P* <0.05, ^**^*P* <0.01, ^***^*P* <0.001 *versus* NTG mice.

Supplemental table S2. Echocardiographic characteristics of WIF1 transgenic mice

(line 17) at 1, 3, 6 and 10 months of age

| age | 1 mo | | 3 mo | | 6 mo | | 10 mo | |
| --- | --- | --- | --- | --- | --- | --- | --- | --- |
| Group | NTG | WIF1 | NTG | WIF1 | NTG | WIF1 | NTG | WIF1 |
| Number | *n* = 24 | *n* = 17 | *n* = 24 | *n* = 16 | *n* = 24 | *n* = 25 | *n* = 24 | *n* = 16 |
| LVEDD, mm | 3.38±0.15 | 3.57±0.15^***^ | 3.69±0.22 | 3.84±0.29 | 3.84±0.21 | 4.21±0.38^***^ | 4.01±0.25 | 4.32±0.48^*^ |
| LVESD, mm | 1.99±0.18 | 2.37±0.18^***^ | 2.37±0.25 | 2.63±0.22^**^ | 2.56±0.24 | 3.00±039^***^ | 2.85±0.25 | 3.10±0.46^*^ |
| LVPWD, mm | 0.46±0.07 | 0.49±0.07 | 0.58±0.06 | 0.60±0.07 | 0.58±0.07 | 0.62±0.08 | 0.57±0.09 | 0.61±0.07 |
| LVPWS, mm | 0.80±0.11 | 0.69±0.07^***^ | 0.89±0.09 | 0.77±0.09^***^ | 0.85±0.15 | 0.80±0.10 | 0.77±0.07 | 0.77±0.07 |
| LVAWD, mm | 0.62±0.14 | 0.61±0.06 | 0.71±0.08 | 0.67±0.07 | 0.75±0.08 | 0.70±0.07^*^ | 0.72±0.10 | 0.70±0.05 |
| LVAWS, mm | 0.99±0.12 | 0.77±0.13^***^ | 1.08±0.13 | 0.87±0.08^***^ | 1.11±0.15 | 0.87±0.10^***^ | 0.92±0.13 | 0.90±0.08 |
| LVEF, % | 72.88±4.63 | 63.33±5.54^***^ | 66.20±5.63 | 59.90±5.55^**^ | 62.20±7.21 | 55.40±7.19^**^ | 55.98±6.03 | 54.68±6.50 |
| LVFS,% | 41.16±3.91 | 33.78±3.91^***^ | 36.07±4.27 | 31.57±3.92^**^ | 33.35±5.50 | 28.76±4.58^**^ | 28.99±3.97 | 28.29±4.02 |
| HR, bpm | 441.67±40.47 | 442.76±49.31 | 420.94±58.62 | 414.13±31.52 | 439.47±49.85 | 428.54±33.94 | 434.98±51.59 | 427.09±34.95 |

LVEDD: left ventricle (LV) end-diastole diameter; LVESD: LV end-systole diameter; LVPWD: LV posterior wall at end-diastole; LVPWS: LV posterior wall at end-systole; LVAWD: LV anterior wall at end-diastole; LVAWS: LV anterior wall at end-systole; LVEF: LV ejection fraction; LVFS: LV fractional shortening; HR: heart rate. ^*^*P* <0.05, ^**^*P* <0.01, ^***^*P* <0.001 *versus* NTG mice.
